# Comparable reduction in Zif268 levels and cytochrome oxidase activity in the retrosplenial cortex following mammillothalamic tract lesions

**DOI:** 10.1016/j.neuroscience.2016.05.030

**Published:** 2016-08-25

**Authors:** Aura Frizzati, Michal M. Milczarek, Frank Sengpiel, Kerrie L. Thomas, Christopher M. Dillingham, Seralynne D. Vann

**Affiliations:** a School of Psychology, Cardiff University, Tower Building, Park Place, Cardiff CF10 3AT, UK; b School of Biosciences, Cardiff University, Museum Avenue, Cardiff CF10 3AX, UK; c Institute of Neuroscience, Trinity College Dublin, Lloyd Building, College Green, Dublin 2, Ireland

**Keywords:** ANOVA, Analysis of variance, AP, antero-posterior, DV, dorso-ventral, LM, lateral-medial, MTT, mammillothalamic tract, Rdg, retrosplenial dysgranular cortex, Rga, retrosplenial granular a cortex, Rgb, retrosplenial granular b cortex, diencephalic amnesia, hippocampus, immediate-early gene, mammillary bodies, memory, anterior thalamic nuclei

## Abstract

Damage to the mammillothalamic tract (MTT) produces memory impairments in both humans and rats, yet it is still not clear why this diencephalic pathway is vital for memory. One suggestion is that it is an important route for midbrain inputs to reach a wider cortical and subcortical network that supports memory. Consistent with this idea, MTT lesions produce widespread hypoactivity in distal brain regions as measured by the immediate-early gene, c-*fos*. To determine whether these findings were selective to c-*fos* or reflected more general changes in neuronal function, we assessed the effects of MTT lesions on the expression of the immediate-early gene protein, Zif268 and the metabolic marker, cytochrome oxidase, in the retrosplenial cortex and hippocampus. The lesions decreased levels of both activity markers in the superficial and deep layers of the retrosplenial cortex in both its granular and dysgranular subregions. In contrast, no significant changes were observed in the hippocampus, despite the MTT-lesioned animals showing marked impairments on T-maze alternation. These findings are consistent with MTT lesions providing important, indirect inputs for normal retrosplenial cortex functioning. These distal functional changes may contribute to the memory impairments observed after MTT lesions.

## Highlights

• Mammillothalamic tract lesions impaired T-maze alternation performance.
• Mammillothalamic tract lesions reduced Zif268 levels in retrosplenial cortex.
• Mammillothalamic tract lesions reduced cytochrome oxidase in retrosplenial cortex.
• No changes were found in the dorsal hippocampus.
• These distal changes may contribute to the memory impairments.

## Introduction

The mammillothalamic tract (MTT) is a white matter bundle, which carries unidirectional projections from the mammillary bodies to the anterior thalamic nuclei. Damage to the MTT appears to be a consistent feature of patients with diencephalic amnesia following stroke (e.g., Van der Werf et al., 2003, Yoneoka et al., 2004, Carlesimo et al., 2007). Furthermore, MTT lesions in rats produce impairments on a number of spatial memory tasks (Field et al., 1978, Thomas and Gash, 1985, Vann and Aggleton, 2003, Vann et al., 2003, Winter et al., 2011, Nelson and Vann, 2014). It is, however, still uncertain why damage to this structure has such notable effects on memory.

One possibility is that disconnecting the mammillary body projections to the anterior thalamic nuclei results in a loss of ascending midbrain inputs, e.g., from Gudden’s tegmental nuclei (e.g., Vann, 2013, Vann and Nelson, 2015), which in turn causes distal hypoactivity in other connected memory structures. This account is consistent with previous findings, as lesions to the MTT or the anterior thalamic nuclei reduce the expression of the immediate early gene *c-fos* in both the retrosplenial cortex and the dorsal hippocampus (Jenkins et al., 2002a, Jenkins et al., 2002b, Jenkins et al., 2004, Poirier and Aggleton, 2009, Vann and Albasser, 2009, Vann, 2013; but see Dupire et al., 2013, Loukavenko et al., 2015).

The present study investigated whether the MTT lesion-induced changes in the hippocampus and retrosplenial cortex are selective to the immediate-early gene c-*fos*, or reflect a more widespread dysfunction. Two different markers of neuronal activity were assessed: Zif268 is a transcription factor involved in the regulation of a number of synaptic proteins and, hence, is implicated in synaptic plasticity (e.g., Davis et al., 2003, Knapska and Kaczmarek, 2004); cytochrome oxidase is a component of the mitochondrial electron transport chain required for oxidative phosphorylation and can be used to map levels of neural metabolism (Wong-Riley, 1989, Poremba et al., 1998). Both markers are, therefore, implicated in neuronal activity but via different pathways. Furthermore, increased levels of c-Fos, Zif268 and cytochrome oxidase have been found in both the retrosplenial cortex and hippocampus of rats performing spatial memory tasks (Vann et al., 2000a, Vann et al., 2000b, Soule et al., 2008, Pothuizen et al., 2009, Barbosa et al., 2013). If the projections carried via the MTT *are* important for the optimal functionality of the retrosplenial cortex and hippocampus during spatial memory performance, it might be expected that MTT lesions would disrupt the expression of all three neuronal markers.

Two separate cohorts of rats with MTT lesions were examined: tissue from the first cohort was stained for the expression of Zif268 while tissue from the second cohort was used to quantify levels of cytochrome oxidase. Animals from both cohorts were tested on a forced-run version of the radial-arm maze task in a novel room prior to perfusion to increase neuronal activity above baseline. The lesions in the second cohort were additionally verified both behaviorally and immunohistochemically, i.e., rats were tested on a reinforced T-maze alternation task and the tissue was processed for calbindin-staining to visualize the dense fibrous stain in the ventrolateral part of the anteroventral thalamic nucleus attributed to mammillothalamic input (e.g., Rogers and Resibois, 1992).

## Experimental procedures

### Animals

Subjects were 43 naïve male Dark Agouti rats (Harlan, Bicester, UK). The 20 rats in Cohort 1 (Zif268) weighed 215–250 g at the time of surgery; the 23 rats in Cohort 2 (cytochrome oxidase) weighed 226–252 g at the time of surgery.

Rats were housed in pairs under diurnal light conditions (14 h light/10 h dark) and behavioral testing was carried out during the light phase at a regular time of day. Rats were thoroughly handled before the study began and were given free access to water throughout the experiments. During the behavioral test period the animals were food deprived but their body weight did not fall below 85% of their free feeding weight. The rats from both cohorts had been part of previously published studies (Vann and Albasser, 2009, Nelson and Vann, 2014). The rats in Cohort 1 had been part of a study assessing the effects of MTT lesions on the expression of another immediate-early gene protein, c-Fos. The rats in Cohort 2 came from a separate behavioral study (Nelson and Vann, 2014) and, as a result, had been tested on several behavioral tasks in addition to those presented here, including a working memory task in the watermaze, an object-in-place task, a go-no-go place discrimination task, a passive placement task in the watermaze and a working memory task in the radial-arm maze. All experiments were carried out in accordance with UK Animals (Scientific Procedures) Act, 1986 and associated guidelines.

### Stereotaxic surgery

Animals in each cohort were divided into two groups: one received bilateral MTT lesions (Cohort 1: MTTx1, *n* = 10; Cohort 2: MTTx2, *n* = 13), while the other group underwent control surgery (Cohort 1: Sham1, *n* = 10; Cohort 2: Sham2, *n* = 10). Before surgery, all animals were deeply anesthetized by intraperitoneal injection of sodium pentobarbital (60 mg/kg pentobarbital sodium salt; Sigma–Aldrich, United Kingdom) and then positioned in a stereotaxic head-holder (David Kopf Instruments, Tujunga, CA, USA). All rats were maintained on oxygen during surgery and given an analgesic (Meloxicam; Boehringer Ingelheim, Rhein, Germany). The position of the incisor bar of the stereotaxic frame was set at +5.0 mm to the interaural line. A midline incision was made on the top of the scalp to expose the dorsal skull, which was drilled at the point of the lesion.

For Cohort 1, an electrode (0.7 mm tip length, 0.25 mm diameter; Radionics TCZ, Radionics, Burlington, VT, USA) was lowered vertically and its tip temperature was raised to 60 °C for 15 s using a RFG4-A Lesion Maker (Radionics). The stereotaxic coordinates were: antero-posterior (AP) −1.2 mm (relative to bregma), lateral-medial (LM) ±0.9 mm (relative to bregma), and dorso-ventral (DV) −6.9 mm (from top of the cortex).

For Cohort 2, an electrode (0.7 mm tip length, 0.25 mm diameter; Diros Technology Inc., Toronto, Canada) was lowered vertically and its tip temperature was raised to 70 °C for 22 s using an OWL Universal RF System URF-3AP lesion maker (Diros Technology Inc.). The stereotaxic coordinates were: AP −2.0 mm (relative to bregma), LM ±0.9 mm (relative to bregma), and DV −6.2 mm (from top of the cortex).

For the surgical controls, the electrode was positioned at the same AP and LM coordinates but was only lowered to a DV position of +1.0 mm above the lesion site to avoid damaging the tract and left *in situ* without raising the temperature of the tip.

After surgery, the skin was sutured, an antibiotic powder applied (Acramide: Dales Pharmaceuticals, UK) and animals received 5 ml of glucose saline subcutaneously. They were then placed in a temperature-controlled recovery box until they awoke from the anesthetic. Animals were allowed 2–3 weeks to recover before starting any behavioral training during which time all animals had recovered their preoperative weight.

### Behavioral testing

#### Standard T-maze task (Cohort 2)

##### Apparatus

Testing was carried out in a modifiable four-arm (cross-shaped) maze. One of the arms could be blocked off to form a T-shaped maze. The floors of the T-maze were made of wood, which had been painted white. Each arm was 45.5 cm long and 12 cm wide. The side-walls (32.5 cm high) were made of black Perspex. At the end of each arm was a sunken food-well (2 cm in diameter and 0.75 cm deep). Access to an arm could be prevented by placing an aluminum barrier at the entrance to the arm. The maze was placed on a table (74 cm high) for the duration of testing. Salient visual cues were hung on the walls of the test room and lighting was provided by overhead lights.

##### Pretraining and testing procedures

Pretraining began 2 months after surgery; prior to this the rats were tested on a watermaze task. Each animal was given 3 days of pretraining such that they would consistently run down the stem of the maze and find sugar pellets (45 mg; Noyes Purified Rodent Diet, Lancaster, NH, USA) in the food-wells in both arms.

After pretraining, the acquisition phase began. Each trial consisted of a forced ‘sample’ phase followed by a free ‘choice’ phase. During the sample phase, one of the goal arms of the T-maze was blocked by an aluminum barrier. After the rat turned into the preselected goal arm, it was allowed to eat one reward pellet, which had previously been placed in the food-well. The rat was then picked up from the maze and immediately returned to the beginning of the start arm, where it was kept for 10 s using another aluminum barrier. Then, the choice phase began: the rat was now allowed to run up the start arm and given a free choice to enter either the left or the right goal arm. The rat received one reward pellet only if it turned in the direction opposite to the forced choice in the sample run (i.e., non-matching to sample), and it was then returned to the holding box. If the animal made an incorrect choice, i.e., returned to the arm visited on the sample run, the rat was confined to that arm for around 5 s before being returned to the traveling box. The rats were tested in groups of four, with each animal having one trial in turn so that the inter-trial interval was approximately 4 min. The animals received eight trials per day for a total of 8 days.

#### Forced runs in a radial-arm maze (Cohorts 1 and 2)

##### Apparatus

The eight-arm radial maze consisted of a wooden central platform (diameter 34 cm) and eight equally spaced wooden radial arms (each 87 cm long and 10 cm wide); the walls of the arms were made of clear Perspex panels (height 12 cm). A clear Perspex guillotine door (height 24 cm) attached to a pulley system was placed at the beginning of each arm so that access from the central platform to each arm could be controlled by the experimenter. There were food-wells at the end of each arm into which the sucrose reward pellets could be placed.

Two identical radial-arm mazes (one for the training and the other for the final test day) were placed in two rooms easily discriminable for size (Room 1: 295 cm × 295 cm × 260 cm; Room 2: 255 cm × 330 cm × 260 cm), shape, lighting and with distinct visual cues on the walls to help animals to orientate in the maze (e.g., high-contrast stimuli and geometric shapes).

##### Training procedures

A forced-run version of the radial arm maze task was used because MTT-lesioned rats are normally impaired on the standard working memory version (Vann and Aggleton, 2003, Nelson and Vann, 2014). In this way, lesioned and control animals could be matched for motor responses and number of rewards received. For Cohort 1, training started 3 weeks after surgery; for Cohort 2 the time between surgery and training was 11 months. As the rats in Cohort 1 were behaviorally naïve they underwent more training sessions on the forced-run radial-arm maze task. The rats in Cohort 2 had previously undergone training on a working memory version of the radial-arm maze task (Nelson and Vann, 2014) and, therefore, only required a couple of days of training.

Rats were trained to retrieve sucrose pellets for running down pre-selected arms of the radial arm maze. All arms were baited at the beginning of each trial (a trial comprises a visit to all eight arms) and access to the arms was controlled by the experimenter operating the guillotine doors through a pulley system. At the end of one trial (when all eight arms had been visited) the rat was placed in a holding box for approximately 2 min; during this time all the arms were re-baited. For each of the training sessions (Cohort 1: 11 days; Cohort 2: 2 days), rats had one session of this behavioral task, lasting 20 min in total and consisting of multiple trials. Within each session, the arm sequences were different and randomized for consecutive trials. For all behavioral sessions, the animals were placed in a dark and quiet room for 30 min before the beginning of the session and for 90 min after testing was completed. On the final test day (day 12 for Cohort 1; day 3 for Cohort 2), animals were tested on the same task but this time in a novel room. Animals were again placed in the dark before and after testing and were perfused 90 min after testing was completed. This time window was chosen as it has been shown this is the time required for Zif268 protein to be maximally expressed after neuronal stimulation (e.g., Richardson et al., 1992, Zangenehpour and Chaudhuri, 2002). The animals from Cohort 2 were also perfused 90 min after behavioral testing to match procedures for both Cohort 1 and those in a previous study looking at the effects of anterior thalamic nuclei lesions on cytochrome oxidase activity (Mendez-Lopez et al., 2013).

### Tissue processing

Animals in both cohorts were anesthetized with sodium pentobarbital (60 mg/kg, Euthatal, Rhone Merieux, UK) and then transcardially perfused with 0.1 M phosphate buffer saline (PBS) followed by 4% paraformaldehyde in PBS (PFA). The brains were then extracted and postfixed in 4% PFA for 4 h, and then transferred to 25% sucrose in distilled water overnight. On the following day, brains were cut in the coronal plane using a freezing microtome (slice thickness 40 μm for Cohort 1 and 30 μm for Cohort 2).

For Cohort 1, two series (1 in 3) of sections were collected in PBS: one was used for Zif268 staining, while the other was mounted directly onto gelatin-coated slides and stained using cresyl violet, a Nissl stain, for verification of the lesion location and size. For Cohort 2, two series of sections were cryoprotected in an ethylene glycol/sucrose solution at −20 °C and subsequently processed either for cytochrome c oxidase staining or for calbindin staining. A third series was mounted directly onto gelatin-coated slides and stained using cresyl violet, a Nissl stain, for verification of the lesion location and size.

#### Zif268 immunohistochemistry

For Zif268 staining, sections were transferred to 10-mM citrate buffer (pH 6.0) dissolved in deionized H_2_O, and then incubated in a water bath at 70 °C for 30 min. Subsequently, sections were incubated for 10 min on a shaker in a solution of 0.3% hydrogen peroxidase (Fisher Scientific, USA) in PBST (0.2% Triton in PBS), in order to block endogenous peroxidase activity. They were then washed four times (10 min each) with PBST. Next, sections were incubated with a primary anti-Zif268 antibody (Egr-1 (C19): Sc-189; Santa Cruz Biotechnology, Texas, USA) diluted 1:3000 in PBST. The sections were stirred on the shaker in the primary antibody solution for 10 min at room temperature and then they were incubated in the same primary antibody solution for 48 h at 4 °C (at the end of the first 24 h the sections in the primary antibody solution were stirred again for another 10 min at room temperature and then returned to the fridge). Sections were then rinsed again four times in PBST (10 min each), and then incubated in biotinylated goat secondary antibody (diluted 1:200 in PBST; Vectastain, Vector Laboratories, Burlingame, USA) and 1.5% normal goat serum, and left on the shaker for 2 h at room temperature. After this, sections were washed four times in PBST (10 min each) and processed with avidin-biotinylated horseradish peroxidase complex in PBST (Vectastain Elite ABC kit PK-6100, Vector Laboratories, UK) for 1 h at room temperature, again with constant rotation on the shaker. Sections were then rinsed four times in PBST (10 min each), and then washed two times (10 min each) in 0.05 M Tris buffer (pH 7.4, prepared diluting Trizma base in distilled water). Finally, sections were incubated with 3,3′-diaminobenzidine (DAB Substrate Kit, Vector Laboratories, UK) until a brown stain was obtained (requiring usually no more than one minute); the reaction was stopped in cold PBS. Sections from MTTx1 and Sham1 animals were processed simultaneously. Sections were mounted on gelatin-coated slides, dehydrated through a graded series of ethanols and cover-slipped (DPX, Thermoscientific, UK).

#### Cytochrome c oxidase staining

The sections were first washed six times in PBS (10 min each) to remove any residual cryoprotectant. The sections were then washed twice in 0.1 M Tris buffer (5 min each). The incubation medium (30 mg DAB (Sigma D-5637), 15 mg cytochrome c (Sigma C2506), 2.4 g sucrose; all in 0.1 M Tris buffer) and the sections were then separately warmed to 37 °C, and then combined and incubated for a further 30–60 min until a desirable level of staining was obtained. The reaction was stopped by washing with 0.1 M Tris buffer three times (5 min each) and sections were stored at 4 °C overnight. The following day, sections were again washed three times 0.1 M Tris buffer (5 min each), mounted, air-dried and cover-slipped. Tissue from the MTTx2 and Sham2 groups was processed together.

#### Calbindin immunohistochemistry

The sections were washed six times in PBS to remove any residual cryoprotectant (10 min each). This was followed by an endogenous peroxidase activity block in 0.3% hydrogen peroxide solution (0.3% H_2_O_2_, 10% methanol, distilled water), after which the sections were washed three times in PBS (10 min each). To minimize non-specific binding, sections were incubated in 3% horse serum in PBST (PBS plus Triton X-100 at 500 μL/1L PBS) for 1 h and then incubated for 48 h with a primary anti-calbindin antibody (D-28 K, CB300, Swant, Switzerland) in 1% horse serum in PBST. Following this incubation period, sections were washed three times in PBST (10 min each) and incubated with a secondary antibody (in 1% horse serum in PBST) raised against the primary antibody molecule for 2 h (BA-2000, Vector Laboratories). This was followed by three PBST washes (10 min each) and incubation in an avidin–biotin-kit solution (Elite Kit, Vector Laboratories) for 1 h. Following four more washes (10 min each), the label was developed with the DAB kit according to the manufacturer’s instructions (DAB Substrate Kit, Vector Laboratories). All incubations took place at room temperature and, except for the final incubation, on a shaker. The sections were then mounted on gelatin-coated glass slides, air-dried and cover-slipped.

### Imaging

Imaging and subsequent analyses were carried out without knowledge of group assignment. Images were obtained with a Leica DMRB microscope used in combination with an Olympus DP70 camera.

#### Zif268-positive cell counts (Cohort 1)

The program ImageJ (1.46r version, NIH, US) was used to convert the images from RGB to 8-bit grayscale and to count the number of Zif268-positive nuclei above threshold (the threshold value was set manually for each image). Montages were made so that the entire region (i.e., hippocampus or retrosplenial cortex) was included in the counting procedure on each coronal section that was assessed. This counting procedure is not stereological and so does not provide absolute Zif268-positive cell numbers; rather, it provides relative numbers, thus permitting group comparisons. All nuclei that were above threshold and within a size range of 10–100 μm^2^ were counted. For each brain region analyzed, Zif268-positive cell counts were taken from an average of six sections (consecutive where possible) per brain and a mean was calculated for each animal.

#### Cytochrome c oxidase densitometry analysis (Cohort 2)

**Fig. 1:**
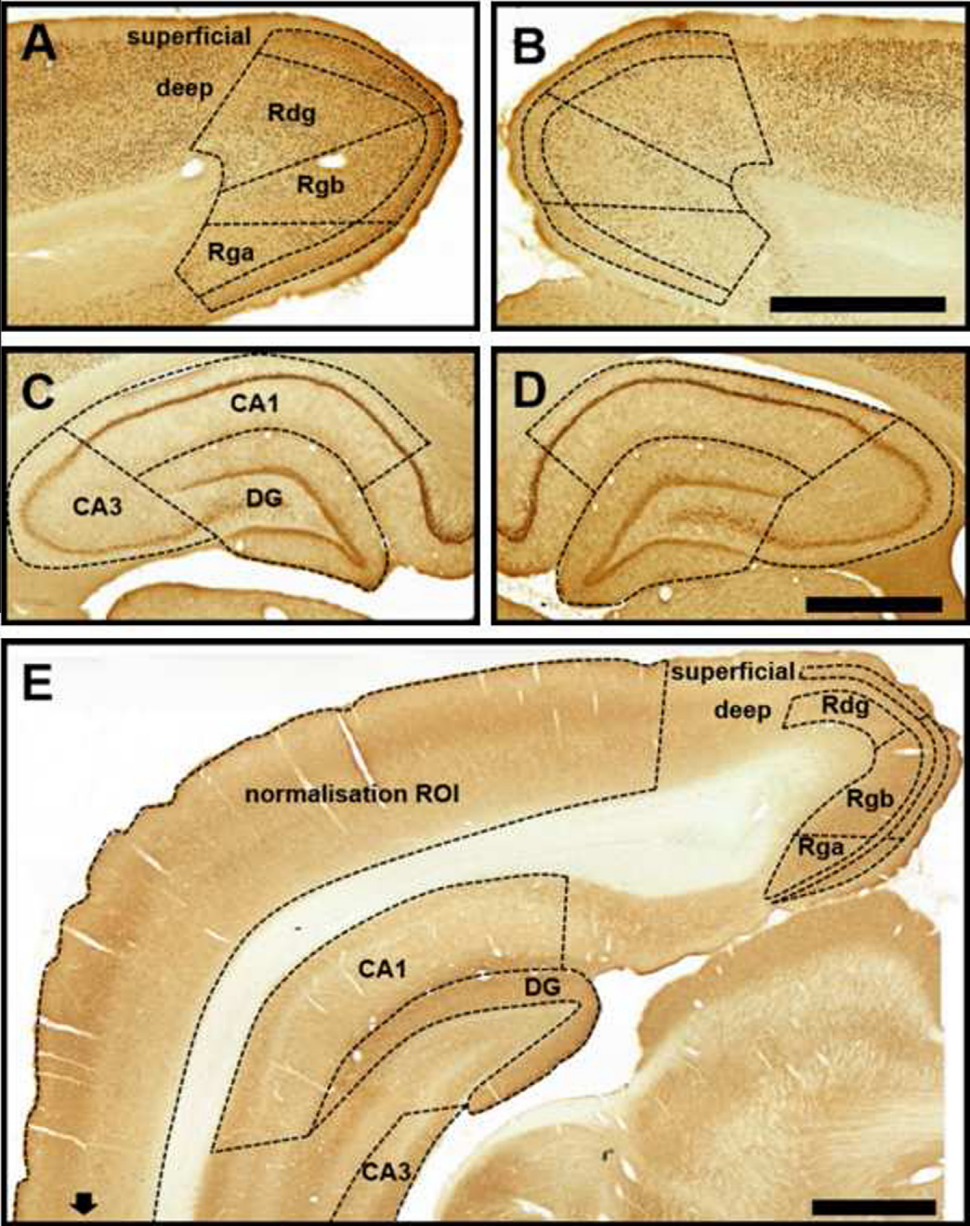
Representative images of Zif268 (A–D) and cytochrome oxidase (E) staining in coronal brain slices. Dashed lines denote the extent of the regions of interest based on Wyss & Van Groen (1992). A, C show examples of anti-Zif268 staining in the retrosplenial cortex and the dorsal hippocampus in a sham brain while B, D display the same regions in a lesioned brain. E – an example of cytochrome oxidase staining comprising both the retrosplenial and the hippocampal regions. Note that the normalization ROI extends beyond the border of the photograph. DG, dentate gyrus; Rga, retrosplenial granular a cortex; Rgb, retrosplenial granular b cortex; Rdg, retrosplenial dysgranular cortex; ROI, region of interest. Scale bar = 1 mm.

For each brain, an average of six coronal sections were imaged in gray scale. Raw values were converted into optical density using a logarithmic function obtained by following guidelines from the National Institute of Mental Health website (Research Services Branch, National Institute of Mental Health, 2014). A normalization procedure was carried out to minimize the effect of different levels of staining between sections, in which values for all regions of interest (as described in the following section) within each hemisphere of a coronal section were divided by the value measured from the remaining cortex (lying outside of the regions of interest) within the same hemisphere of the coronal section (see Fig. 1E). As nonspecific background staining would give a value of 1, this value was subtracted from all normalized values so the final values represent a change from baseline. For each region of interest, within each subject, a single mean normalized value was calculated that represented the level of cytochrome oxidase activity, relative to the rest of the cortex.

### Regions of interest

The regions of interest were identified in coronal sections (Fig. 1). In the dorsal hippocampus, measurements were made within the three main subfields, i.e., dentate gyrus, CA1, and CA3, at ∼−2.56 mm from bregma for Cohort 1 and ∼−4.2 mm to −6.0 mm from bregma for Cohort 2. The Zif268 counts were taken from the pyramidal layers of all hippocampal subfields, while the cytochrome oxidase measurements included all layers of CA1 and CA3 and the molecular layer of the dentate gyrus.

For the retrosplenial cortex, measures were taken from the caudal regions so that all three main subregions were present on the same coronal section. The three main subregions included granular b (Rgb), granular a (Rga), and dysgranular (Rdg) cortex (Wyss & Van Groen 1992). For Zif268, separate counts were taken for the superficial (II and upper III) and deep (lower III to VI) layers of the retrosplenial cortex, as determined by an abrupt change in cell size and packing density (Jenkins et al., 2004, Vann and Albasser, 2009). Finally, cytochrome oxidase measurements in the retrosplenial cortex were acquired from layer II (superficial) and layers V–VI (deep), so that a direct comparison could be made with a recent study by Mendez-Lopez et al. (2013). The identification/verification of the boundaries of these superficial and deep layers was aided by their noticeably darker staining.

### Statistical analysis

All statistical analyses were carried out using SPSS software (version 20, IBM Corporation). The alpha level was set at *p* < 0.05.

### Behavior

#### T-maze alternation (Cohort 2)

An analysis of T-maze performance for Cohort 2 was carried out using the mean number of correct arm choices over blocks of two days; results were compared using a mixed repeated-measures ANOVA with the between-subject factor Lesion (i.e., MTTx versus Shams) and the within-subject factor Block.

#### Zif268-positive cell counts (Cohort 1) and Cytochrome c oxidase densitometry (Cohort 2)

Analyses were carried out either on mean counts of Zif268-positive nuclei for Cohort 1, or on normalized mean cytochrome oxidase optical density (O.D.) values for Cohort 2; in both cases, the means were derived by averaging the values obtained for all the sections for each region of interest in each brain. Means from Cohort 1 and Cohort 2 were analyzed separately using a mixed ANOVA design; different anatomical areas were grouped together, so that each ANOVA comprised related brain areas.

Analysis of the retrosplenial cortex was carried out with Lesion as between-subject factor (two levels: MTTx/Shams), while Region (three levels: Rga/Rgb/Rdg) and Layer (two levels: superficial/deep) were the within-subject factors.

Analysis of the dorsal hippocampus was carried out with Lesion as a between-subject factor (two levels: MTTx/Shams), and Region as a within-subject factor (three levels: dentate gyrus/CA3/CA1).

When the sphericity assumption was violated, a Greenhouse–Geisser correction was applied to the degrees of freedom. When significant interactions were found, the simple effects for each brain region were analyzed as recommended by Winer (1971) using the pooled error term. Significant three-way interactions were followed up using multiple comparisons with a Bonferroni correction. The main effect of Region (and Layer in the case of the retrosplenial cortex) was not considered meaningful due to inherent differences between regions (e.g., staining levels and region size) but interactions with Lesion group are reported.

## Results

### Histological assessment of lesion success

**Fig. 2:**
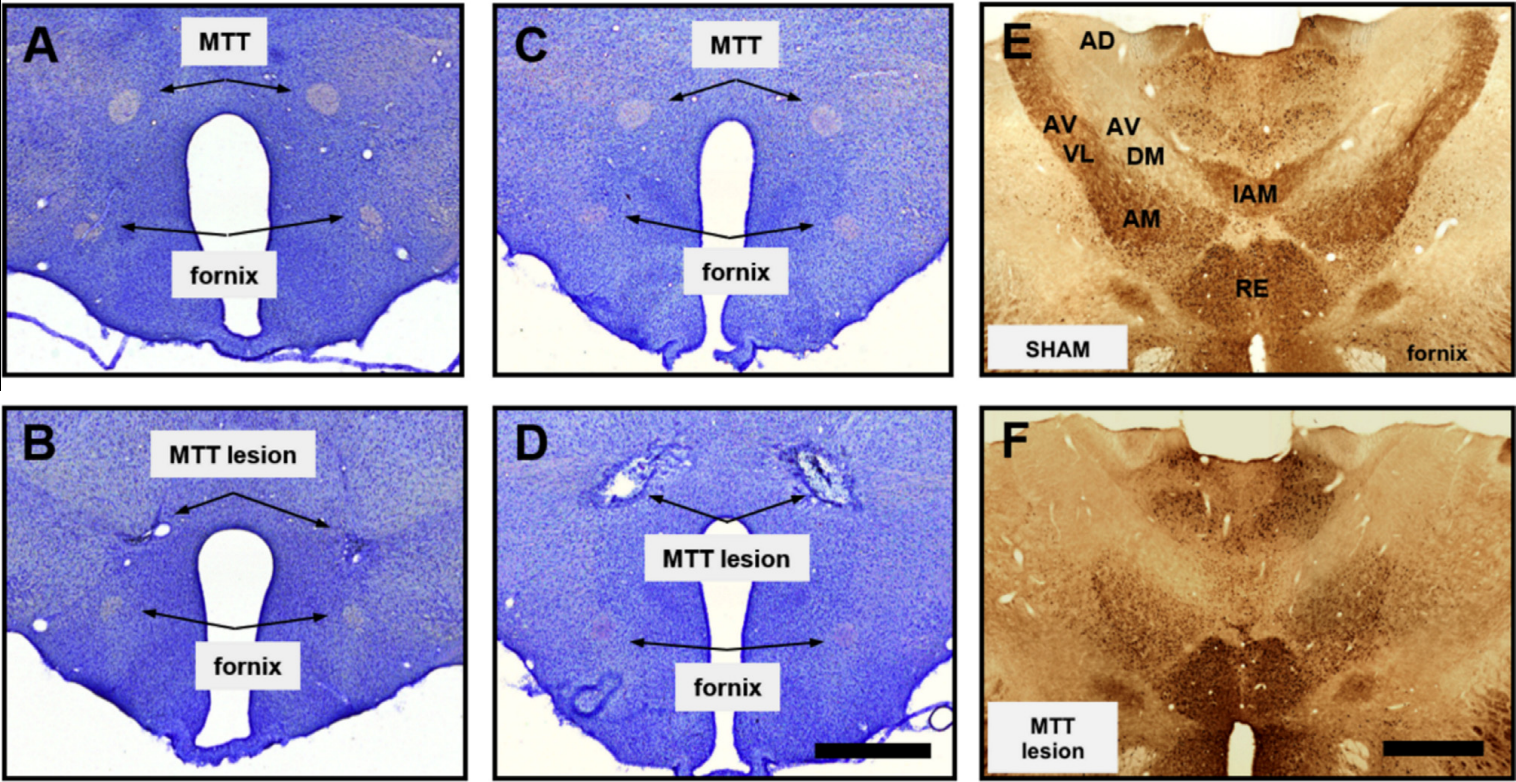
Histological confirmation of mammillothalamic tract lesions. Images A–D show cresyl violet staining of sham (A, C) and lesioned brains (B, D) in Cohort 1 (A, B) and Cohort 2 (C, D). The mammillothalamic tract (MTT) is absent in the lesioned brains while the fornix remains intact. E,  F show anti-calbindin immunostaining in the anterior thalamus of a sham brain (E) and a lesioned brain (F) from Cohort 2. The dark neuropil stain of the ventrolateral portion of the anteroventral thalamic nucleus (AVVL) is absent following mammillothalamic tract lesions whereas staining in the anteromedial (AM) and reuniens (RE) nuclei remains unaffected. AD, anterodorsal nucleus; AVDM, dorsomedial anteroventral nucleus; IAM, interanteromedial nucleus. Scale bar = 1 mm.

*Cohort 1* – Based on histological verification of lesion sites using cresyl violet stained sections, nine out of ten rats displayed discrete and complete bilateral MTT lesions (Fig. 2A, B), while one rat had a partial disconnection of the tract in the left hemisphere. These MTT lesions have been shown to selectively disconnect the projections from the medial mammillary nuclei to the anterior medial and anterior ventral thalamic nuclei (Vann and Albasser, 2009). Given the medial mammillary body – anterior thalamic nuclei projections are unilateral (Hayakawa and Zyo, 1989), Zif268-positive cell counts were carried out in the right hemisphere only of the one case with partial unilateral sparing.

*Cohort* 2 – Based on cresyl violet staining, ten out of thirteen rats received complete bilateral lesions while the remaining three had bilateral sparing and so were removed from subsequent analyses (Fig. 2C, D). The results from the calbindin staining aligned with the observations from the cresyl violet staining. Consistent with the calbindin-positive neuropil staining in the anterior thalamic nuclei being attributed to the MTT inputs, lesioned animals showed a complete loss of the marker in the ventrolateral part of the anteroventral thalamic nucleus as would be expected with a complete tract disconnection (Fig. 2E, F). There was also a loss of fibrous staining in the interanteromedial thalamic nucleus. In contrast, the cell body staining for calbindin, most noticeable in the anteromedial thalamic nucleus, was unaffected by the lesion.

### Reinforced T-maze alternation (Cohort 2)

**Fig. 3:**
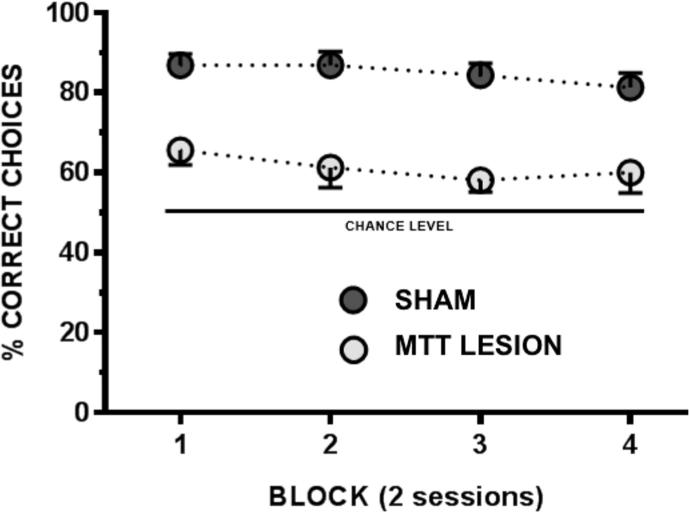
T-maze spatial alternation performance in Cohort 2 animals. Each block comprises two sessions made up of eight trials. The horizontal line denotes chance, i.e., 50%. Error bars represent standard error of the mean.

The efficacy of the lesions in Cohort 2 was also confirmed behaviorally, using reinforced T-maze alternation. The lesions significantly impaired performance of this task (*F*_(1,18)_ = 67.13, *p* < 0.001) (Fig. 3); this impairment persisted across training as reflected by a lack of effect of Block or Block by Lesion interaction (both *F* < 1).

### Zif268-positive cells (Cohort 1)

#### Dorsal hippocampus

**Fig. 4:**
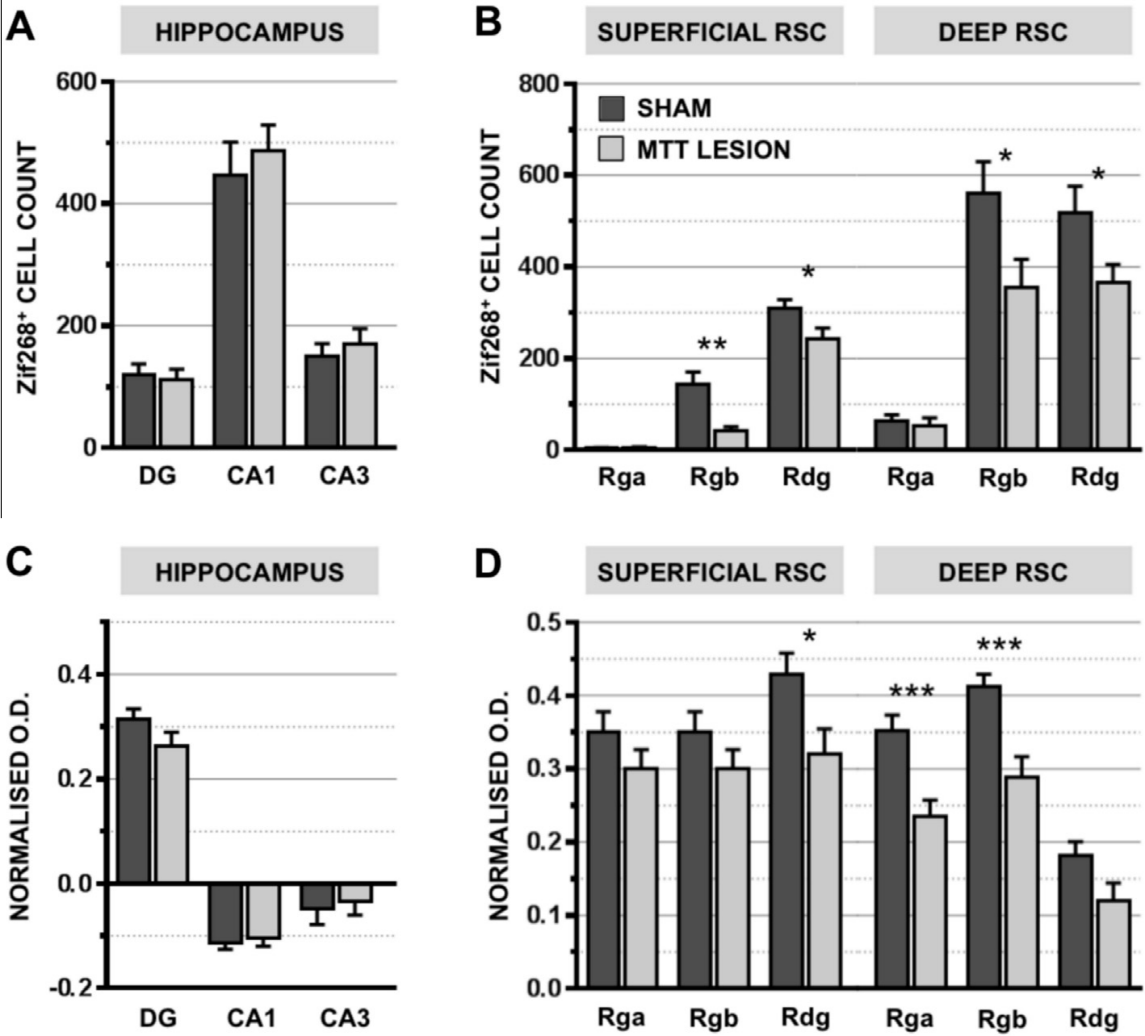
Zif268 cell counts from Cohort 1 (A, B) and cytochrome oxidase optical density (O.D.) measures from Cohort 2 (C, D) coronal brain slices for the hippocampus (A, C) and the retrosplenial cortex (B, D) regions. Regions of interest in the hippocampus are: the dentate gyrus (DG), CA1 and CA3; in the retrosplenial cortex: the Rga (retrosplenial granular a), Rgb (retrosplenial granular b) and Rdg (retrosplenial dysgranular) further subdivided into superficial (SUPERFICIAL RSC) and deep (DEEP RSC) areas. ^*^p < 0.05, ^**^p < 0.01, ^***^p < 0.001, Error bars = standard error of the mean.

MTT lesions were found to have no significant effect on Zif268 expression in the dorsal hippocampus, as reflected by the lack of effect of Lesion and Lesion by Region interaction (both *F* < 1; Fig. 4A).

#### Retrosplenial cortex

The MTT lesions significantly reduced Zif268 cell counts in the retrosplenial cortex (*F*_(1,18)_ = 5.7, *p* < 0.05; Figs. 1A, B and 4B). The effect of lesion was dependent on the region analyzed, as demonstrated by the significant Lesion × Region interaction (*F*_(2,36)_ = 6.0, *p* < 0.01). Analysis of the simple effects revealed significant differences in the Rgb and Rdg subregions, (superficial Rgb: *p* < 0.01; deep Rgb *p* < 0.05; superficial Rdg: *p* < 0.05; deep Rdg: *p* < 0.05). There was no difference in Rga, however, the counts in this subregion were typically very low, potentially producing a floor effect.

### Cytochrome oxidase staining (Cohort 2)

Within the gray matter, the cytochrome oxidase DAB reaction produced dark staining of the neuropil and, to a lesser extent, cell bodies. The retrosplenial cortex showed alternating bands of high and low intensity, which clearly delineated the borders between layers I, II, III–IV and V–VI; and generally a higher level of staining compared to the adjacent regions of the secondary visual cortex and the dorsal subiculum (Fig. 1E). The layers of the hippocampus also displayed an alternating pattern of high and low intensity staining with strong label in stratum oriens of CA1 and CA3 and the molecular layer of the dentate gyrus (Fig. 1E).

#### Dorsal hippocampus

The MTT lesions did not affect overall hippocampal cytochrome oxidase staining (*F* < 1) and there were no significant regional differences in the effect of lesion (*F*_(1.52, 27.37)_ = 3.30, *p* = 0.064) (Fig. 4C).

#### Retrosplenial cortex

In the retrosplenial cortex, MTT lesions produced an overall reduction in cytochrome oxidase staining, reflected by a main effect of Lesion (*F*_(1,18)_ = 8.11, *p* = 0.011). Furthermore, there was a significant three-way Lesion-by-Region-by-Layer interaction (*F*_(2,36)_ = 15.91, *p* < 0.001). Subsequent analyses revealed significant decreases in the deep layers of the granular retrosplenial cortex (Rga and Rgb both *p* < 0.001) and the superficial dysgranular layer (*p* = 0.027; Fig. 4D).

## Discussion

MTT lesions produce memory impairments in both rodents and humans, yet it is still not clear why damage within this region is so disruptive. In the present set of experiments, MTT lesions in rats reduced activity in the retrosplenial cortex, as measured by expression levels of the immediate-early gene *zif268* and the metabolic marker cytochrome oxidase. These changes were found both in the superficial and deep layers of the granular and dysgranular retrosplenial subregions. In contrast, no significant changes were found in the dorsal hippocampus. Despite these markers being assessed in two separate cohorts of MTT-lesioned rats, the pattern of findings across both markers was remarkably similar, consistent with changes in both markers reflecting underlying hypometabolism within the retrosplenial cortex. These findings show the importance of ascending mammillary projections for retrosplenial function and also show that retrosplenial dysfunction does not simply reflect thalamic deafferentation, given the MTT lesions would only indirectly affect the retrosplenial cortex.

The changes in levels of Zif268 and cytochrome oxidase can be compared to the expression of c-Fos, which was assessed using the same cohort of rats that was used to measure Zif268 expression in the present experiment (Vann and Albasser, 2009). MTT lesions resulted in more pronounced changes in retrosplenial c-Fos expression, especially in the more superficial layers; for example, c-Fos expression was reduced by about 70% in the superficial layers of granular b (Vann and Albasser, 2009) compared to a 27% reduction in Zif268 and a 14% reduction in cytochrome oxidase. The degree of change in the deeper retrosplenial layers was more comparable across the three different markers. However, in contrast to the present Zif268 and cytochrome oxidase findings, the MTT lesions also reduced c-Fos expression in the dorsal hippocampus (see also Vann, 2013). Similar to the results found with c-Fos, MTT lesions (and more general diencephalic lesions) can also reduce levels of hippocampal acetylcholinesterase (Savage et al., 2003, Winter et al., 2011). Therefore, it seems that although retrosplenial hypoactivity following MTT lesions appears constant across markers, the hippocampal effects are much more varied; some markers are reduced across all hippocampal subfields while other measures of activity indicate that the hippocampus is functionally “normal”, despite animals showing clear memory deficits. The effect of diencephalic lesions on hippocampal function is clearly multifaceted and may well reflect both the activity marker under investigation (e.g., Barry et al., 2015) and the sensitivity of the behavioral task.

The MTT carries projections from the mammillary bodies to the anterior thalamic nuclei and nearly all neurons within the mammillary bodies are thought to contribute fibers to the MTT (Cruce, 1975, Takeuchi et al., 1985, Vann et al., 2007). The MTT lesions in the present study disconnected the projections from the medial mammillary nuclei to the anteromedial and anteroventral thalamic nuclei while leaving the projections from the lateral mammillary nuclei largely intact (Vann and Albasser, 2009). The use of anti-calbindin immunohistochemistry in Cohort 2 of the present study also confirmed that the medial mammillary projections to the anterior thalamic nuclei had been successfully disconnected. It has been proposed that there are at least two separate systems within the mammillary bodies, comprising the lateral and medial nuclei (Dillingham et al., 2015). The lateral mammillary nuclei form part of a head-direction system whereas the medial mammillary nuclei are part of a proposed “theta” system (Vann and Aggleton, 2004, Hopkins, 2005, Vann, 2010). It appears that the medial mammillary nuclei are more important for mnemonic function, with lateral mammillary nuclei lesions producing only mild and transient impairments on tests of spatial memory (Vann, 2005, Vann, 2011, Harland et al., 2015).

Given that the MTT lesion effects are most likely mediated via the anterior thalamic nuclei, and the anteroventral and anteromedial thalamic nuclei in particular, comparisons with findings from anterior thalamic lesions become especially pertinent. The effects of both unilateral and bilateral anterior thalamic lesions on retrosplenial and hippocampal activity have been assessed using a number of markers of activity, including c-Fos, Zif268 and cytochrome oxidase. Overall, the pattern is remarkably similar to what is found with MTT lesions. Expression of c-Fos is typically reduced in both the retrosplenial cortex and hippocampus (Jenkins et al., 2002a, Jenkins et al., 2002b, Jenkins et al., 2004, Poirier and Aggleton, 2009; but see Dupire et al., 2013, Loukavenko et al., 2015), whereas Zif268 changes are restricted to the retrosplenial cortex (Poirier and Aggleton, 2009, Dumont et al., 2012). In the same way, anterior thalamic lesions reduce levels of cytochrome oxidase in the retrosplenial cortex but not in the hippocampus (van Groen et al., 1993, Mendez-Lopez et al., 2013). Given the similarity in changes following anterior thalamic and MTT lesions, it would appear that the anterior thalamic lesion effects are primarily being driven by their ascending inputs from the mammillary bodies; in contrast, fornix lesions that disconnect the hippocampal projections to the anterior thalamic nuclei and mammillary bodies have far less pronounced effects on retrosplenial immediate-early gene expression (Vann et al., 2000c).

In the present experiment, there was a significant decrease in cytochrome oxidase expression both in the superficial and deep layers of dysgranular retrosplenial cortex and the deep layers of granular retrosplenial cortex. In contrast, an earlier study assessing cytochrome oxidase activity after bilateral anterior thalamic lesions only found a decrease in layer 2 of retrosplenial granular b (Mendez-Lopez et al., 2013). The apparent differences between the findings from anterior thalamic and MTT lesions may reflect indirect versus direct loss of innervations, given that changes in cytochrome oxidase can reflect transsynaptic disconnection (Wong-Riley et al., 1978, Wong-Riley and Riley, 1983). Alternatively, methodological differences, e.g., time post-surgery, the extent to which the lesions encompass the anteromedial and anteroventral thalamic nuclei, and the length and type of behavioral training used, may have contributed to the different pattern of changes. There is some evidence that retrosplenial hypoactivity is affected by the post-surgery interval in anterior thalamic-lesioned animals, with only superficial granular b being affected initially and more widespread hypoactivity, including deeper granular layers and dysgranular cortex, found months after surgery (e.g., Jenkins et al., 2004). However, a separate study found widespread retrosplenial changes a month after surgery (Poirier and Aggleton, 2009), i.e., within the same time-frame used in the Mendez-Lopez et al. Study (2013). Time since surgery appears to have very little effect when considering the distal effects of MTT lesions as very similar patterns of c-Fos changes are found both 3 weeks and 9 months post-surgery (Vann and Albasser, 2009, Vann, 2013). To determine whether there is a genuine difference between MTT and anterior thalamic nuclei lesions in the time-frame of changes to the deeper retrosplenial layers, the lesion effects would need to be compared directly within the same study.

The retrosplenial cortex hypoactivity following MTT and anterior thalamic lesions appears to be a robust finding across markers of activity. Furthermore, pathology within the medial diencephalon in patients has also been also associated with retrosplenial hypoactivity (Joyce et al., 1994, Reed et al., 2003). In rats, the hypoactivity does not appear to be task dependent as changes have been found across a number of behavioral tasks and retrosplenial hypoactivity (as measured by c-Fos) has even been reported in home-cage animals with anterior thalamic lesions (Jenkins et al., 2002b), perhaps reflecting an underlying hypometabolic state. To understand how retrosplenial cortex dysfunction may contribute to subsequent spatial memory impairments, a comparison of lesion effects becomes particularly relevant. In rats, MTT and retrosplenial lesions typically produce very similar profiles of impairment, with the clearest deficits found on spatial memory tasks (Vann and Aggleton, 2002, Vann and Aggleton, 2003). While both structures appear important for path integration (Cooper and Mizumori, 1999, Winter et al., 2011), the spatial memory impairments cannot simply be attributed to an inability to navigate; retrosplenial cortex lesions and MTT lesions impair performance on a location discrimination task and an object-in-place task, which both tax aspects of spatial memory but lack a navigational component (Hindley et al., 2014, Nelson and Vann, 2014). To date, there have been no direct comparisons of animals with retrosplenial and MTT lesions within the same study; however, when comparing across studies, it would appear that on certain tasks, MTT or mammillary body lesions can be more disruptive than retrosplenial cortex lesions (Parker and Gaffan, 1997, Pothuizen et al., 2008, Vann, 2013, Buckley and Mitchell, 2016). Thus, it is unlikely that retrosplenial dysfunction alone is sufficient to explain all MTT-lesion induced memory impairments but it may, nevertheless, exacerbate the lesion effects. The retrosplenial cortex appears to be particularly sensitive to damage within the Papez circuit and is one of the first brain regions to show hypoactivity in Mild Cognitive Impairment and early Alzheimer’s disease (e.g., Minoshima et al., 1997). Understanding why this structure is so severely affected by distal pathology and whether this hypoactivity contributes to any cognitive impairments will be the goal of future research.
